# Cyanidin-3-O-Glucoside improves the viability of human islet cells treated with amylin or Aβ_1-42_ *in vitro*

**DOI:** 10.1371/journal.pone.0258208

**Published:** 2021-10-06

**Authors:** Jennifer Croden, Josue Rodrigues Silva, Wenlong Huang, Nancy Gupta, Wen Fu, Kaja Matovinovic, Mazzen Black, Xian Li, Kunsong Chen, Yulian Wu, Jack Jhamandas, Gina R. Rayat

**Affiliations:** 1 Department of Surgery, Ray Rajotte Surgical-Medical Research Institute, Alberta Diabetes Institute, Faculty of Medicine and Dentistry, University of Alberta, Edmonton, Alberta, Canada; 2 Division of General Surgery, The First Affiliated Hospital of Shantou University Medical College, Shantou, Guangdong, China; 3 Department of Medicine (Neurology) and the Neuroscience Mental Health Institute, Faculty of Medicine and Dentistry, University of Alberta, Edmonton, Alberta, Canada; 4 Department of Horticulture, College of Agriculture and Biotechnology, Zhejiang University, Hangzhou, China; 5 Department of Surgery, The Second Affiliated Hospital of Zhejiang University, Hangzhou, China; Tohoku University, JAPAN

## Abstract

Islet transplantation is being considered as an alternative treatment for type 1 diabetes. Despite recent progress, transplant recipients continue to experience progressive loss of insulin independence. Cyanidin-3-O-Glucoside (C3G) has shown to be protective against damage that may lead to post-transplant islet loss. In this study, human islets cultured with or without C3G were treated with human amylin, Aβ_1-42_, H_2_O_2_, or rapamycin to mimic stresses encountered in the post-transplant environment. Samples of these islets were collected and assayed to determine C3G’s effect on cell viability and function, reactive oxygen species (ROS), oxidative stress, amyloid formation, and the presence of inflammatory as well as autophagic markers. C3G treatment of human islets exposed to either amylin or Aβ_1-42_ increased cell viability (p<0.01) and inhibited amyloid formation (p<0.01). A reduction in ROS and an increase in HO-1 gene expression as well as *in vitro* islet function were also observed in C3G-treated islets exposed to amylin or Aβ_1-42_, although not significantly. Additionally, treatment with C3G resulted in a significant reduction in the protein expression of inflammatory markers IL-1β and NLRP3 (p<0.01) as well as an increase in LC3 autophagic marker (p<0.05) in human islets treated with amylin, Aβ_1-42_, rapamycin, or H_2_O_2_. Thus, C3G appears to have a multi-faceted protective effect on human islets *in vitro*, possibly through its anti-oxidant property and alteration of inflammatory as well as autophagic pathways.

## Introduction

Islet transplantation is being considered as a possible treatment for patients with type 1 diabetes. Despite recent progress in the field, transplant recipients continue to experience a progressive loss of insulin independence. Inflammatory damage [1], creation of reactive oxygen species (ROS) [2], amyloid plaque formation [3–5], and toxicity from the immunosuppressive drugs that are required to prevent rejection of the islet graft post-transplantation [6] have all been suggested as potential causes of transplant failure; however, the precise reason(s) remains poorly understood.

Studies examining the cause of islet transplant failure have identified amyloid formation as a significant contributing factor [7]. Studies in which human islets were transplanted into the liver of patients with type 1 diabetes [8, 9] and under the kidney capsule or into the liver and spleen of immune-deficient mice [3, 10–15] have shown subsequent amyloid deposition and β-cell death. Amyloids are insoluble aggregates of proteins generated through highly abnormal or aberrant folding processes [16] and are formed within the islets of humans, cats, and monkeys, but not rodents or pigs [14, 17]. Formation and deposition of amyloidogenic proteins is the pathological hallmark of type 2 diabetes and Alzheimer’s disease, conditions in which islet amyloid polypeptide (also called amylin) is deposited in the pancreas and amyloid β (Aβ) in the brain, respectively.

Amylin is a hormone that is found in all mammals studied to date [18] and is co-stored and co-secreted with insulin [19–21]. Existing evidences suggest that amylin aggregation induces processes that lead to the impairment of β-cell function and death thus contributing to the loss of islet β-cell mass [22–24]. One such process could be the disruption of mitochondrial membrane by the amyloid aggregates, thereby causing metabolic dysfunction with induction of oxidative stress and ROS production, and consequently damage to islet cells [25, 26]. Oxidative stress can lead to an intracellular accumulation of autophagosomes, which overloads the cell and subsequently induces cell death [27, 28]. Thus, the prevention of amyloid formation or attenuation of its toxicity through reduction of oxidative stress could potentially prolong the survival of human islet transplants. It has been observed that amylin and Aβ_1-42_ are both toxic to neurons when applied *in vitro* [29, 30]. Although amylin is known to have detrimental effect on human islets [25, 26], it is not clear whether Aβ_1-42_ has a similar effect on human islets.

Previous studies have shown that flavonoids could protect pancreatic β-cells against oxidative stress-induced damage [31, 32]. Anthocyanins, a type of flavonoid, are naturally occurring polyphenolic compounds in plant foods [32] and are the most abundant water-soluble pigments present in fruits and vegetables [33]. Berry extract rich in anthocyanins has been linked to the protective effects observed in some disease models [34], including diabetes [35]. Such protective effects of anthocyanins are attributed to their typical chemical structures, which contain many aromatic rings, hydroxyl groups and various glycosylation sites [35]. The natural electron deficiency of anthocyanins endows these compounds with high reactivity toward ROS, making them among the most powerful natural antioxidants in dietary plants. We showed that treatment with cyanidin-3-O-glucoside (C3G), a major anthocyanin found in the extract of Chinese bayberry (*Myrica rubra*) [36], could enhance the function of syngeneic mouse islet grafts [37]. We also reported that C3G protected neonatal porcine islets from harmful effects of ROS and enhanced their function in diabetic mice [38]. It is conceivable that C3G may be beneficial to human islets by protecting them against oxidative stress-induced damage, as well as preventing the formation and deposition of amyloid thereby enhancing their function.

## Materials and methods

### Human islet isolation

Human islets were obtained from the Alberta Diabetes Institute Human Islet Core Laboratory of the University of Alberta. The donors’ next of kin under University of Alberta ethics approval # Pro00013094 provided informed consent. Islets were prepared and cultured following published protocol [39, 40]. Purity of the human islets was determined using 0.5 mg/mL dithizone (Sigma-Aldrich, Ontario, Canada) in Hank’s Balanced Salt Solution (HBSS, Sigma-Aldrich, Ontario, Canada) containing 2% fetal calf serum. Islets used in this study were obtained from five female (age 42 to 68, BMI: 21.1 to 35.4) and nine male (age 23 to 61, BMI: 22.7 to 36.6) donors and could not be used for transplantation into human patients. Detailed information about the human donors and the preparation of human islets can be found at www.isletcore.ca.

### Preparation of C3G

Chinese bayberry fruits (“Biqi” cultivar) containing high amounts of C3G were harvested from an orchard owned by Xianju County (Lat. 28°51’ N, 120°44’ E), Zhejiang Province, China. This location was chosen because Chinese bayberry fruit, commercially popular nation-wide in China, develops very well in Xianju County. The Chinese bayberry fruits were acquired with permissions from the owner abiding by the respective laws in China and were paid through the Zhejiang University project financial budget. The fruit samples were botanically authenticated by Dr. Changjie Xu from the Fruit Science Institute of Zhejiang University (Zhejiang, China). Fruits were harvested at commercial maturity from orchards of Xianju County in June 2016 and transported to the lab of Zhejiang University in Hangzhou within 6 hours after harvest. The fruit were frozen by liquid nitrogen immediately without storage. The fruits in uniform shape and color without disease and mechanical damage were selected and frozen in liquid nitrogen. The pulp was then ground into a fine powder and stored at -80°C for further experiments. The fruit extract was prepared as follows: the frozen fruit samples were extracted in methanol acidified with 0.5% acid. Sugar and other impurities were removed by solid phase extraction to get the fruit extracts. The content of C3G was measured and found to be 54.12%. A full description of the fruit extract preparation can be found in a publication by Yan et al [41], who prepared the samples used in these experiments. The compound was then confirmed by high-performance liquid chromatography (HPLC) analysis with comparison to standard substance and LC-MS identification, following the published protocol by Schieber et al [42]. Briefly, an HPLC system (2695 pump, 2996 diode array detector, Waters) coupled with an ODS C18 analytical column (4.6 × 250 mm) was used for characterizing individual flavonoid compounds. The column was operated at a temperature of 25°C and the compounds were detected between 200 and 600nm. The mobile phase of HPLC consisted of 0.1% (v/v) formic acid in water (eluent A) and of acetonitrile: 0.1% formic acid (1:1, v/v) (eluent B). The gradient program was as follows: 0–40 minutes, 10–38% of B; 40–60 minutes, 38–48% of B; 60–70 minutes, 48–100% of B; 70–75 minutes, 100–10% of B; 75–80 minutes, 10% of B. The prepared fruit extract was diluted with sterile double distilled water to a concentration of 50μM as the working stock solution.

### Treatment of human islets

One thousand islet equivalents (IEQ) in 1mL of CMRL 1066 media (Fisher Scientific, New Hampshire, USA) supplemented with 0.5% BSA (Sigma-Aldrich, Ontario, Canada), 1% insulin-transferrin-selenium (Fisher Scientific), 1% L-glutamine (Invitrogen, California, USA), 100U/mL penicillin, and 100µg/mL streptomycin were placed in each well of two non-treated 6-well culture plates (BD Biosciences, Ontario, Canada). Five wells were designated as untreated (without C3G) and five wells were designated as treated with 1μM C3G. The islets were incubated for 24 hours at 22°C, 5% CO_2_, and 95% air. Ten-μM human amylin (American Peptide, California, USA), 10μM soluble oligomeric Aβ_1-42_ (rPeptide, Georgia, USA) prepared as previously described [29], or 10µM rapamycin (Sigma-Aldrich) were then added to 1 untreated well and 1 C3G-treated well and the islets were incubated for further 24 hours. At the 22-hour period of incubation, 1mM of hydrogen peroxide (H_2_O_2_) was added to 1 untreated and 1 C3G-treated well and the islets were incubated for the remaining 2 hours.

### Insulin and glucagon staining

Zinc formalin-fixed islets that were stored in 70% ethanol were washed three times in PBS. The islets were then permeabilized with 0.25% Triton X-100 (Fisher Scientific) and treated with 10% methanol in H_2_O_2_ (Fisher Scientific). Islet samples were treated with 20% normal goat serum, and then incubated with polyclonal guinea pig anti-porcine insulin (1:1,000 dilution, DAKO, Ontario, Canada, Cat. No. A0564) or monoclonal mouse anti-porcine glucagon (1:5,000 dilution, Sigma-Aldrich, Cat. No. G2654) primary antibodies. After washing, biotinylated goat anti-guinea pig IgG (1:200 dilution, Vector Laboratories, Ontario, Canada, Cat. No. BA7000) and biotinylated goat anti-mouse IgG (1:200 dilution, Jackson ImmunoResearch Laboratories Inc., Pennsylvania, USA, Cat. No. 115065166) secondary antibodies were added to the respective samples. Avidin-biotin complex/horseradish peroxidase (ABC/HP, Vector Laboratories) and 3,3-diaminobenzidinetetrahydrochloride (DAB, BioGenex, California, USA) were used to produce a brown color for a positive enzyme and substrate reaction. Samples were counter-stained with Harris’ hematoxylin (Electron Microscopy Sciences, Pennsylvania, USA) and eosin (Sigma-Aldrich) before visualization by light microscopy.

### Cell viability, Thioflavin-S and ROS amyloid assays

Fifty IEQ from each treatment group were collected and centrifuged for 1.5 minutes at 1,000 rpm and the supernatant was removed. For viability assay, samples from 4–5 islet preparations were used. Islet pellets were suspended in 2μM calcein and 4μM ethidium homodimer solution for 30 minutes at 37°C, 5% CO_2_, and 95% air (physiological conditions). For ROS assay, islet samples from 5–6 preparations were suspended in dichlorodihydrofluorescein diacetate solution (Abcam, Ontario, Canada) and incubated for 30 minutes at physiological conditions. After incubation, islets were washed in PBS and were placed on glass microscope slides then fixed with 4% paraformaldehyde. Islets were then counterstained with 30μM DAPI (Fisher Scientific) to visualize cell nuclei before 50µL of 50% glycerol was added to the slide and the islets were covered with a coverslip. The sample slides were kept frozen at -20°C until analyzed by immunofluorescence microscopy. For Thioflavin-S assay, islet samples from 5–6 preparations were suspended in PBS and placed on slides. The sample slides were immersed in 100mL of solution containing 0.0125g of Thioflavin-S (Sigma-Aldrich) and 50% ethanol for 5 minutes, washed with 50% ethanol and then with distilled water. Islets were then counterstained with 30μM DAPI then 50µL of 50% glycerol was added to the slide and the islets were covered with a coverslip. The sample slides were kept frozen at -20°C until analyzed by immunofluorescence microscopy.

### Immunofluorescence staining for LC3, NLRP3 and IL-1β

Islets on glass microscope slides were treated with 4% paraformaldehyde (Fisher Scientific) and rinsed with PBS. The islets were then permeabilized with 0.5% Triton X-100 and binding of non-specific proteins were blocked with 5% FBS in PBS. Polyclonal rabbit anti-human IL-1β (Abcam, Cat. No. AB2105), polyclonal rabbit anti-human LC3 (Sigma-Aldrich, Cat No. L8918), and monoclonal rat anti-human NLRP3 (R&D Systems, Minnesota, USA, Cat. No. MAB7578) primary antibodies were used to identify the specific molecules that these antibodies recognize. This step was followed by washing the islet samples with PBS and polyclonal Texas Red-conjugated goat anti-rabbit IgG (Vector Laboratories, Cat. No. TI1000) or monoclonal FITC-conjugated mouse anti-rat IgG2a (BD Biosciences, Ontario, Canada, Cat. No. 553896) secondary antibody was added to the samples, respectively. Islet samples were washed in PBS and prepared for immunofluorescence microscopy analysis. In these experiments, islet samples from 3–5 preparations were used.

A Zeiss Photomicroscope III microscope (ZEISS Microscopy, Jena, Germany) was used for fluorescence microscopy to obtain images of the islets. Slides were set under 4x magnification to identify the islets. Once identified, the islets were visualized under 40x magnification. The depth of each islet was set in the best possible view to include most of its structures and a good resolution. Images were captured in different channels (e.g., blue, green, red), while keeping the depth constant. Images were saved in .tif format for later analysis. For each experiment, images were taken from 5–7 different islets in every condition. Quantification of LC3, NLRP3 and IL-1β was performed using Fiji software. Images were split into RGB channels to separate the cellular component to be analyzed. First, the number of cells within the human islets were quantified by counting the DAPI positive cells in the blue channel. Second, to analyze the cellular marker expression, the Fast Fourier Transform (FFT) bandpass function was applied to the green or red channel for background correction. Third, complex masking was used to remove the binary image from the new FFT transformed image. Next, the remaining particles were counted using a gated filter fixed to 1–10 pixels. Finally, the particles counted were divided by the cell number inside the islet and the quotient was used as the relative amount of the cellular component in the image. Consistent treatment of all images allowed for comparison between amounts of the measured cellular component in the control and treatment groups.

### Static glucose stimulation assay

One hundred IEQ, from each treatment group were placed in duplicate individual wells of a non-tissue culture treated 24 well plate that contained RPMI medium alone or RPMI medium with 2.8mM glucose or 20mM glucose (n = 5). Islet samples were incubated for 2 hours at physiological conditions. Without disturbing the islets, 700μL of solution from each well was then placed into a cuvette and analyzed using a Multi-Array Assay System Human Insulin Kit (Meso Scale Discovery, Maryland, USA) to determine the amount of insulin secreted by the beta cells during glucose stimulation.

### Transmission electron microscopy (TEM)

The ultrastructure of islets was examined by TEM. Islet samples were collected and treated with a modified Karnovsky’s solution containing 2% paraformaldehyde and 2% glutaraldehyde in 0.1M sodium cacodylate. The islets were centrifuged at increasing speed to produce a firm cell pellet (1x1 mm-size). Secondary fixation was done in 1% osmiun tetroxide, followed by staining with 1% uranyl acetate in water. The pellets were dehydrated in increasing concentrations of ethanol and transferred into Epon 814 blocks for polymerization. Sectioning was performed with UltracutE (Reichert Jung, New York, USA) followed by imaging using Mega View III Soft Imaging System (Olympus Soft Imaging Solutions, Münster, Germany) with a digital camera mounted on a Philips 410 TEM (FEI Company, Oregon, USA).

### Real time-PCR assay

The total RNA from 200 IEQ of human islets isolated from four male donors (age 48–58 years old, BMI 22.7 to 36.6, purity 75–95%) was extracted using Trizol reagent (Invitrogen) following the manufacturer’s protocol and the concentration of RNA was measured by NanoDrop (ThermoFisher, Ontario, Canada). Two hundred nanograms of total RNA were used to construct cDNA using 5X All-In-One RT MasterMix (Applied Biological Materials, British Columbia, Canada). Gene sequences for human HO-1 (n = 3), LC3 (n = 4), and IL-1β (n = 4) were obtained from Gene Bank in PubMed and primers were designed using Custom Assay Design Tool in Taqman RT-PCR Assays (ThermoFisher). PCR amplifications were performed on a 7900HT Fast Real-Time PCR System (Applied Biosystems, California, USA) using TaqMan gene expression assays (Applied Biosystems).

### ELISA

IL-1β cytokine was measured from the islet supernatants using sandwich ELISA following the manufacturer’s protocol (n = 4). Briefly, Nunc-Immuno 96-well plates were coated overnight (at 4°C) with purified anti-human IL-1β antibody at 1μg/mL in DPBS (BioLegend, California, USA, Cat. No. 511601). The next day, plates were blocked with DPBS + 1% BSA for 2 hours at 25°C, and then samples and standards were added to the 96-well plate in 2 replicates and incubated for 2 hours at 25°C. A dilution of 1:2 was used for the samples with the standards ranging from 20 to 5000pg/mL (BioLegend, Cat. No. 579409). Plates were subsequently incubated with biotin-conjugated antibody at 2μg/mL for 1 hour at 25°C (BioLegend, Cat. No. 511703), followed by incubation with Streptavidin-alkaline phosphatase (1:2000 dilution, BD Biosciences, Cat. No. 554065) for 1 hour at 25°C. Plates were washed 3 times with 1xDPBS containing 0.05% Tween-20 after each incubation step. Color was developed by adding PNPP substrate (Sigma-Aldrich) and absorbance was detected at 405nm using SpectraMAX 250 spectrophotometer (Molecular Devices Corp., California, USA). The amount of IL-1β in each experimental sample was determined from the absorbance value plotted against the concentration of a standard curve for the cytokine.

### Statistical analysis

All experiments were performed at least in triplicate and the data were presented as means ± standard deviation. Statistical analysis was performed with the GraphPad Prism 9.1.1 software. Differences were examined using application of one-way ANOVA or Student’s t-test. A p-value of 0.05 or less was considered statistically significant.

## Results

### Human islets following isolation and culture

**Fig 1:**
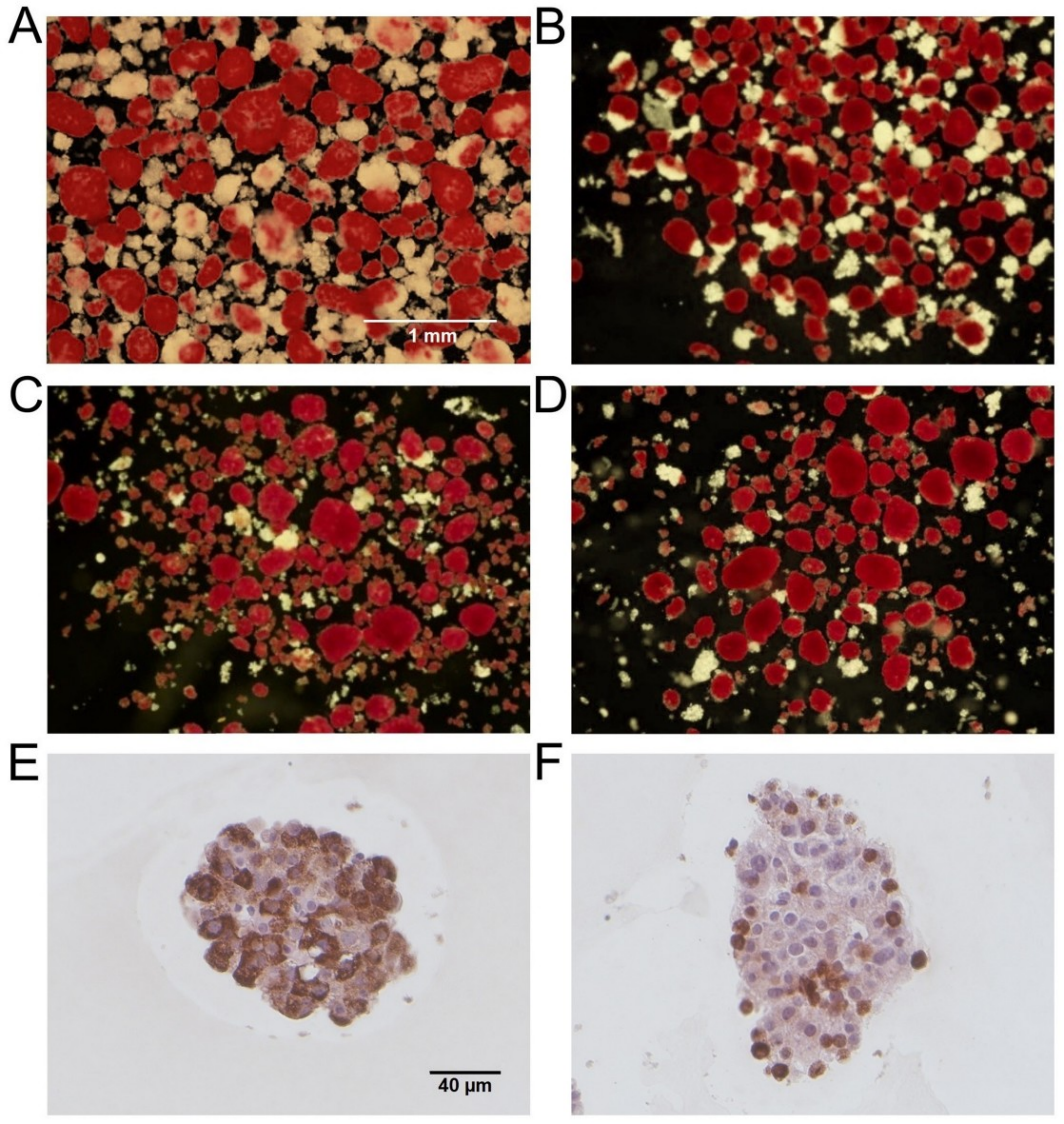
Representative human islet preparations. Representative dithizone-stained images of human islets used in the study with purity of 75% (A, n = 2), 80% (B, n = 3), 90% (C, n = 5), and 95% (D, n = 4). Dithizone-stained endocrine cells (red) and non-dithizone-stained exocrine cells (white) are shown. Scale bar represents 1mm. Human islets stained for insulin (E) and glucagon (F), counterstained with Harris’ hematoxylin and eosin. Scale bar represents 40µm.

Human islets used in this study demonstrate robust gross morphology with varying degrees of contaminating acinar tissues following isolation and culture (Fig 1A–1D). The purity of islets was 75% (n = 2, Fig 1A), 80% (n = 3, Fig 1B), 90% (n = 5, Fig 1C), and 95% (n = 4, Fig 1D). Immunostaining showed the presence of insulin-positive beta cells (Fig 1E) and glucagon-positive alpha cells (Fig 1F) in the human islets.

### C3G increased the viability of human islets exposed to amylin or Aβ_1-42_

**Fig 2:**
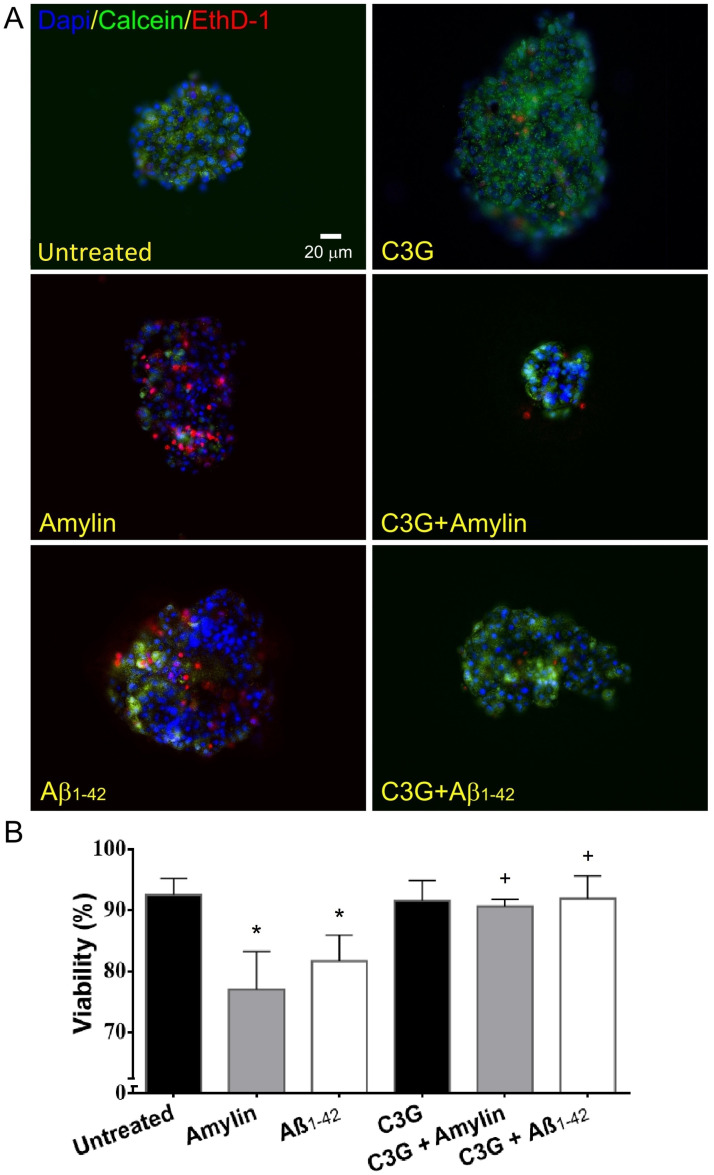
Viability of human islets treated with or without C3G and with or without amylin or Aβ_1-42_. (A) Islets stained with calcein (green, live), ethidium (red, dead), and DAPI (blue, nuclei). Representative untreated, amylin-treated, Aβ_1-42_-treated, C3G-treated, C3G + amylin-treated, C3G + Aβ_1-42_-treated islets are shown. Scale bar represents 20μm. (B) Percentage of viability in untreated or C3G-treated islets exposed to amylin or Aβ_1-42_ (*p<0.001, untreated vs. amylin; *p<0.01, untreated vs. Aβ_1-42_; n = 4–5; ^+^p<0.05, amylin vs. C3G + amylin; ^+^p<0.05, Aβ_1-42_ vs. C3G + Aβ_1-42_; n = 4).

Islets that were exposed to amylin or Aβ_1-42_ showed a significant decrease (77.1%±6.3% and 81.7%±4.3%, n = 4, respectively, Fig 2A and 2B) in the cell viability compared to islets that were not exposed to either amylin or Aβ_1-42_ (93.0%±2.6%, n = 5). The cell viability observed in untreated islets and islets that were treated with C3G (92.5%±3.6%, n = 5) was comparable. C3G-treated islets that were exposed to amylin demonstrated a significant increase in the cell viability (90.6%±1.2%, n = 4, Fig 2A and 2B) compared to amylin-treated islets without C3G treatment. C3G-treated islets that were exposed to Aβ_1-42_ also showed a significant increase in the cell viability (91.9%±3.7%, n = 4, Fig 2A and 2B) compared to the islets treated with Aβ_1-42_ without C3G.

### C3G inhibited amyloid formation in human islets treated with amylin or Aβ_1-42_

**Fig 3:**
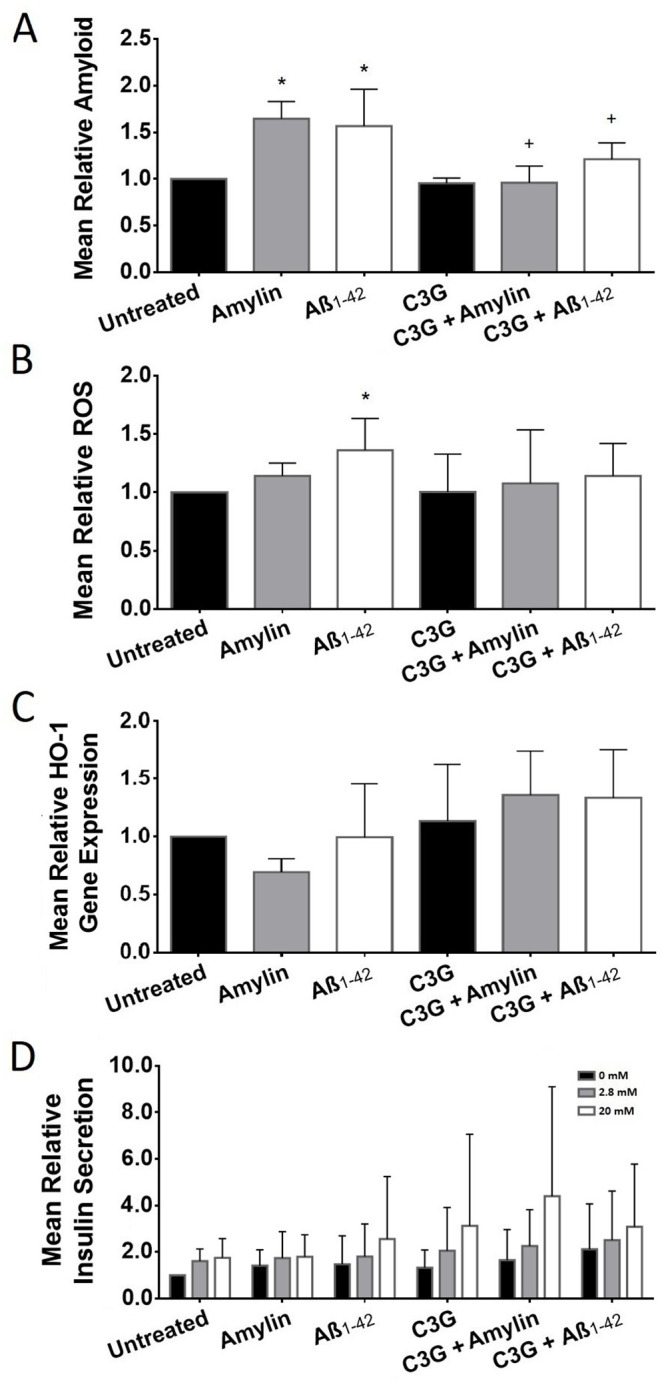
Amyloid formation, ROS production, HO-1 gene expression, and insulin secretion in human islets treated with or without C3G and with or without amylin or Aβ_1-42_. (A) Mean relative amyloid formation in untreated and C3G-treated islets exposed to amylin or Aβ_1-42_ (*p<0.001, untreated vs. amylin or Aβ_1-42_; n = 5–6; ^+^p<0.01, amylin vs. C3G + amylin; ^+^p<0.05, Aβ_1-42_ vs. C3G + Aβ_1-42_; n = 5). (B) Mean relative ROS detected in untreated or C3G-treated islets exposed to amylin or Aβ_1-42_ (*p<0.05, untreated vs. Aβ_1-42_; n = 5–6). (C) Mean relative HO-1 gene expression in untreated or C3G-treated human islets (n = 3). (D) Mean relative *in vitro* static glucose-stimulated insulin secretion of C3G-treated human islets exposed to amylin or Aβ_1-42_ in low glucose (2.8mM, gray bar) and high glucose (20mM, white bar)-containing media. Black bar represents 0mM glucose-containing media (n = 5).

The amyloid formation detected in islets treated with either amylin or Aβ_1-42_ was significantly increased (1.65±0.18 and 1.57±0.40, respectively, n = 5, Fig 3A) compared to the untreated islets (1.00±0.00, n = 6). The amyloid observed in untreated and C3G-treated islets was comparable (0.95±0.06 vs. 1.00±0.00, n = 6). C3G-treated islets exposed to amylin (0.96±0.18, n = 5) showed significantly less amyloid formation compared to the islets that were exposed to amylin without C3G. Likewise, the levels of amyloid detected in C3G-treated islets that were exposed to Aβ_1-42_ (1.21±0.17, n = 5) was significantly less than what was observed in islets treated with Aβ_1-42_ without C3G.

### C3G decreased ROS and increased HO-1 gene expression and insulin secretion treated with amylin or Aβ_1-42_

Islets treated with amylin or Aβ_1-42_ showed an increase (1.14±0.11 and 1.36±0.27, n = 5, respectively, Fig 3B) in the levels of ROS compared to the untreated islets (1.00±0.00, n = 6). The levels of ROS observed in C3G-treated (1.00±0.32, n = 6) and untreated islets were comparable. C3G-treated islets exposed to amylin (1.08±0.46, n = 5) or Aβ _1–42_ (1.14±0.28, n = 5) showed a decreasing trend in the levels of ROS compared to islets exposed to amylin or Aβ_1-42_ without C3G.

Since previously we detected high gene expression of heme-oxygenase-1 (HO-1) in C3G-treated mouse [37] and pig [38] islets exposed to ROS, we also measured the gene expression of HO-1 in human islets treated with C3G after exposure to the stress induced by amylin or Aβ_1-42_ to determine whether C3G could also trigger the upregulation of HO-1 gene expression when exposed to these stressors. In general, HO-1 gene expression appears to be upregulated in islets treated with C3G when compared to untreated islets and those that were exposed to amylin or Aβ_1-42_ (Fig 3C). Islets that were treated with C3G had higher HO-1 gene expression (1.13±0.49, n = 3) compared to the untreated islets (1.00±0.00, n = 3). Islets that were treated with C3G and exposed to amylin (1.36±0.38, n = 3) had higher HO-1 gene expression compared to islets treated with amylin (0.70±0.11, n = 3). In addition, C3G treated islets exposed to Aβ_1-42_ (1.34±0.42) had an increased HO-1 gene expression compared to islets treated with Aβ_1-42_ (0.99±0.46, n = 3).

We were also interested in determining the effect of C3G on insulin secretion upon glucose stimulation of human islets *in vitro*. The amount of insulin released by islets placed in low glucose (2.8mM) and high glucose (20mM)-containing media was measured. C3G-treated islets (n = 5) incubated in media with 20mM glucose demonstrated a 1.8-fold increase in insulin secretion compared to untreated islets (n = 5) incubated in the same condition (Fig 3D). C3G-treated islets that were exposed to amylin and incubated in media with 20mM glucose demonstrated an approximately 2.5-fold increase in insulin secretion compared to untreated islets exposed to amylin. Likewise, C3G-treated islets that were exposed to Aβ_1-42_ and incubated in media with high glucose showed a 1.2-fold increase in insulin secretion compared to untreated islets exposed to Aβ_1-42_ and incubated in media with high glucose.

### C3G enhanced the formation of autophagic vesicles

**Fig 4:**
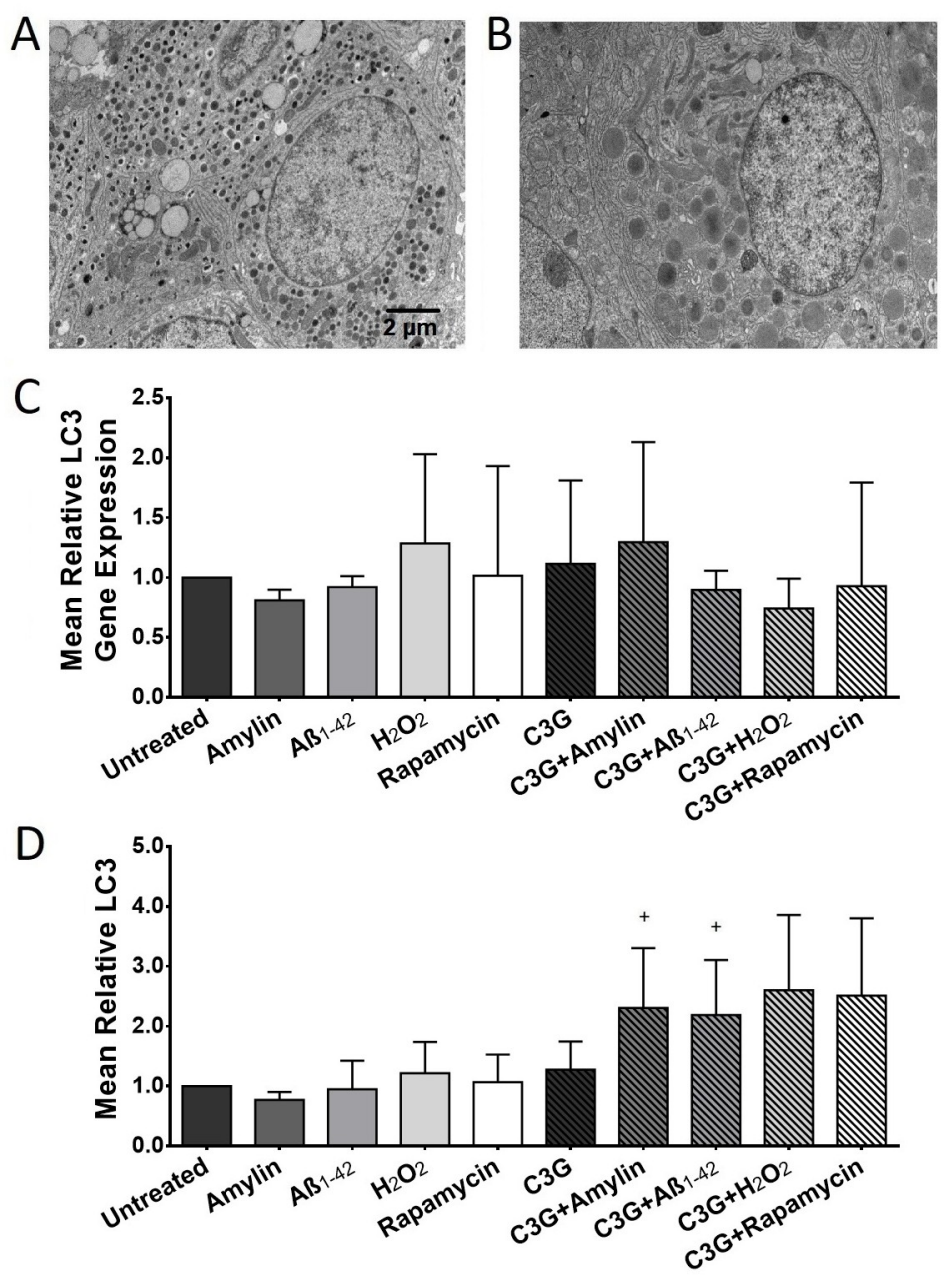
Effect of C3G on autophagy in human islets exposed to amylin, Aβ_1-42_, H_2_O_2_ or rapamycin. (A) Representative TEM image of a β cell in untreated and (B) C3G-treated islets. Scale bar represents 2μm. (C) Mean relative autophagic vesicle marker LC3 gene expression in untreated or C3G-treated human islets exposed to amylin or Aβ_1-42_ (n = 4). (D) Mean relative LC3 protein expression in untreated and C3G-treated human islets exposed to amylin or Aβ_1-42_ (^+^p<0.05, amylin vs. C3G + amylin; ^+^p<0.01, Aβ_1-42_ vs. C3G + Aβ_1-42_; n = 3–5).

The formation of autophagosomes was examined to gain insight into a potential mechanism of C3G’s protective effects against cellular stress *in vitro*. TEM examination of C3G-treated islets (Fig 4B) revealed the presence of more autophagosomes compared to the untreated islets (Fig 4A). LC3 gene expression in C3G-treated islets was higher (1.12±0.70, n = 4, Fig 4C) compared to LC3 gene expression detected in untreated islets (1.00±0.00, n = 4). Since LC3 is a marker for autophagy, we determined the gene and protein expression of LC3 in islets. The gene expression of LC3 observed in C3G-treated islets exposed to amylin was also higher (1.29±0.84, n = 4) compared to islets exposed to the same stressor (0.81±0.09, n = 4). The LC3 gene expression detected in islets treated with Aβ_1-42_ with and without C3G was almost the same (0.90±0.16 vs. 0.92±0.09, respectively). We also examined the LC3 gene expression on islets treated with H_2_O_2_ and rapamycin with or without C3G treatment. Treatment of islets with H_2_O_2_ resulted in increased LC3 gene expression (1.29±0.75, n = 4) compared to the untreated islets. The LC3 gene expression in islets treated with rapamycin was comparable (1.02±0.92, n = 4) to what was observed in the untreated islets. The LC3 gene expression in islets treated with C3G then exposed to H_2_O_2_ or rapamycin was reduced (0.74±0.25, n = 4) or comparable (0.93±0.87, n = 4), respectively when compared to islets treated with H_2_O_2_ or rapamycin only.

The LC3 protein expression observed in C3G-treated islets was higher (1.27±0.47, n = 5, Fig 4D) compared to those detected in the untreated islets (1.00±0.00, n = 5). Islets treated with amylin alone showed a decrease in LC3 levels (0.77±0.13, n = 5) compared to the untreated islets. In contrast, C3G-treated islets that were exposed to amylin showed a significant increase in LC3 protein levels (2.31±1.00, n = 5) compared to the islets exposed to amylin alone. The LC3 protein levels detected in islets treated with Aβ_1-42_ was comparable (0.95±0.48, n = 5) to what was observed in the untreated islets. Islets treated with C3G and Aβ_1-42_ showed significantly higher levels of LC3 (2.19±0.92, n = 5) compared to islets treated with Aβ_1-42_ without C3G. The LC3 protein expression observed in islets treated with H_2_O_2_ was higher (1.22±0.52, n = 3, Fig 4D) compared to the untreated islets. This trend was also observed in C3G-treated islets that were exposed to H_2_O_2_, (2.60±1.26, n = 3) when compared with islets treated with H_2_O_2_ only. Treatment of islets with C3G and rapamycin increased the levels of LC3 detected (2.51±1.29, n = 3) compared to the rapamycin-treated islets (1.07±0.46, n = 3).

### C3G decreased the protein expression of inflammatory markers in human islets

**Fig 5:**
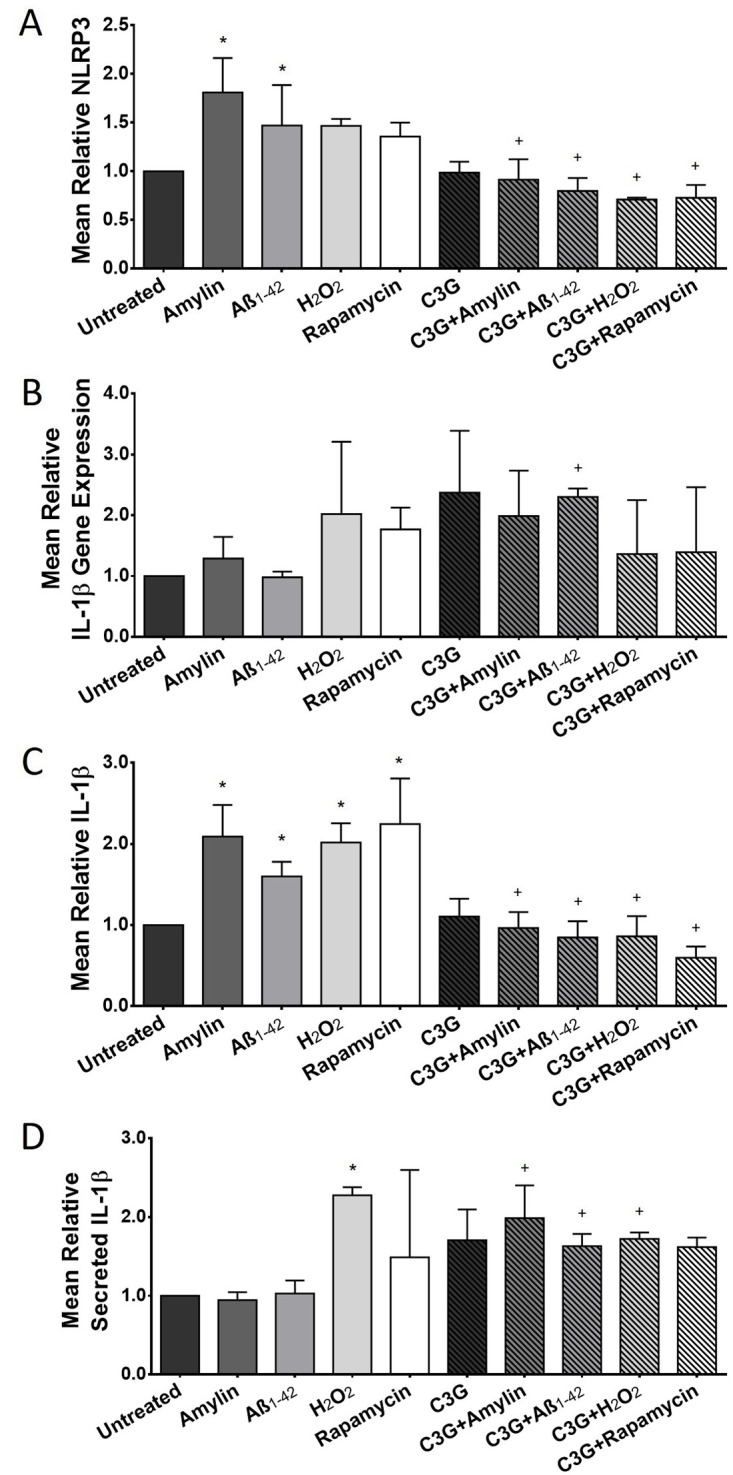
Expression of inflammatory markers in human islets treated with or without C3G and with or without amylin, Aβ_1-42_, H_2_O_2_ or rapamycin. (A) Mean relative NLRP3 protein expression in untreated and C3G-treated islets exposed to amylin, Aβ_1-42_, H_2_O_2_, or rapamycin (*p<0.001, untreated vs. amylin; *p<0.05, untreated vs. Aβ_1-42_; ^+^p<0.01, amylin vs. C3G + amylin; ^+^p<0.05, Aβ_1-42_ vs. C3G + Aβ_1-42_; ^+^p<0.01, H_2_O_2_ vs. C3G + H_2_O_2_; ^+^p<0.05, rapamycin vs. C3G + rapamycin; n = 3–5). (B) Mean relative IL-1β gene expression in untreated and C3G-treated islets exposed to amylin, Aβ_1-42_, H_2_O_2_, and rapamycin n = 3–4). (C) Mean relative IL-1β protein expression in untreated and C3G-treated islets exposed to amylin, Aβ_1-42_, H_2_O_2_, or rapamycin (*p<0.0001, untreated vs. amylin; *p<0.01, untreated vs. Aβ_1-42_; *p<0.001, untreated vs. H_2_O_2_; *p<0.0001, untreated vs. rapamycin; ^+^p<0.001, amylin vs. C3G + amylin; ^+^p<0.001, Aβ_1-42_ vs. C3G + Aβ_1-42_; ^+^p<0.001, H_2_O_2_ vs. C3G + H_2_O_2_; ^+^p<0.05, rapamycin vs. C3G + rapamycin; n = 3–5). (D) Mean relative IL-1β protein detected in culture media of untreated and C3G-treated islets exposed to amylin, Aβ_1-42_, H_2_O_2_, or rapamycin (*p<0.01, untreated vs. H_2_O_2_; ^+^p<0.05, amylin vs. C3G + amylin; ^+^p = 0.05, Aβ_1-42_ vs. C3G + Aβ_1-42_; ^+^p<0.01, H_2_O_2_ vs. C3G + H_2_O_2_; n = 4).

The effect of C3G on islet inflammation was also evaluated. Islets exposed to amylin or Aβ_1-42_ showed significantly higher protein expression of NLRP3 inflammasome (1.81±0.35 and 1.47±0.42, n = 5, respectively, Fig 5A) compared to the untreated islets (1.00±0.00, n = 5). Likewise, the NLRP3 protein expression observed in islets that were exposed to H_2_O_2_ or rapamycin was also increased (1.47±0.07 and 1.36±0.14, n = 3, respectively) compared to the untreated islets. C3G-treated islets that were exposed to amylin (0.91±0.21, n = 5) or Aβ_1-42_ (0.80±0.13, n = 4) showed significantly less NLRP3 compared to the islets exposed to amylin or Aβ_1-42_ without C3G. Similarly, the protein levels of NLRP3 in C3G-treated islets that were exposed to H_2_O_2_ (0.71±0.02, n = 3) or rapamycin (0.73±0.13, n = 3) was also significantly less compared to the islets treated with H_2_O_2_ or rapamycin without C3G. The NLRP3 protein expression detected in C3G-treated islets was comparable (0.99±0.11, n = 5) to those observed in the untreated islets.

The IL-1β cytokine gene expression in islets that were treated with C3G was higher (2.37±1.01, n = 4) compared to those detected in the untreated islets (1.00±0.00, n = 4, Fig 5B). Likewise, the gene expression of IL-β in islets treated with C3G and exposed to amylin (1.99±0.75, n = 4) or Aβ_1-42_ (2.30±0.14, n = 3) was higher compared to the islets exposed to amylin (1.29±0.35, n = 4) or Aβ_1-42_ (0.98±0.09, n = 4). In contrast, the IL-β gene expression was lower in islets treated with C3G and were exposed to H_2_O_2_ (1.37±0.89, n = 4) or rapamycin (1.39±1.07, n = 3) compared to the islets exposed to the same stressors without C3G treatment (2.02±1.19, n = 4 and 1.77±0.36, n = 3, respectively, Fig 5B).

The levels of IL-1β protein expression in islets treated with amylin or Aβ_1-42_ (2.09±0.39 and 1.60±0.18, n = 5, respectively, Fig 5C) were significantly higher compared to the untreated islets (1.00±0.00, n = 5). In contrast, the IL-1β protein detected in C3G-treated islets that were exposed to amylin (0.96±0.20, n = 5) or Aβ_1-42_ (0.84±0.2, n = 5) was significantly less compared to the islets treated with amylin or Aβ_1-42_ without C3G. Treatment of islets with C3G alone did not significantly alter the IL-1β protein expression (1.11±0.22, n = 5) when compared to the untreated islets. Similar to what was observed in NLRP3, the IL-1β protein expression in islets treated with H_2_O_2_ (2.01±0.23, n = 3) or rapamycin (2.24±0.56, n = 3) was significantly higher compared to the untreated islets. The opposite was observed in C3G-treated islets that were exposed to H_2_O_2_ or rapamycin. There was significantly less IL-1β in islets that were treated with C3G and H_2_O_2_ (0.86±0.25, n = 3) or rapamycin (0.60±0.14, n = 3) compared to the islets treated with H_2_O_2_ or rapamycin alone.

The amount of IL-1β secreted in the culture media by the untreated or C3G-treated human islets were also quantified using an ELISA. C3G-treated islets had higher IL-1β compared to the amount detected in the untreated islets (1.71±0.39 vs. 1.00±0.00, respectively, n = 4, Fig 5D). The amount of IL-1β detected in the culture media of islets treated with C3G plus amylin (1.98±0.42, n = 4) or Aβ_1-42_ (1.63±0.16, n = 4), was significantly higher compared to the amount detected in islets treated with amylin or Aβ_1-42_ without C3G (0.94±0.10, and 1.03±0.17, n = 4, respectively). There was also significantly more IL-1β detected in H_2_O_2_-treated islets (2.28±0.10, n = 4) compared to the untreated islets. The amount of IL-β detected in islets treated with C3G and H_2_O_2_ was significantly lower (1.72±0.08, n = 4) compared to the amount detected in islets treated with H_2_O_2_ without C3G. The IL-β observed in islets treated with C3G and rapamycin (1.62±0.12, n = 4) was higher compared to those observed in islets treated with rapamycin without C3G (1.49±1.11, n = 4).

## Discussion

Understanding the mechanism of islet transplant failure remains a major focus of diabetes research in hopes of achieving lifelong insulin independence in transplant recipients. Studies have shown that oxidative stress, inflammatory damage, and amyloid plaque formation likely all play a role in the failure of islet allografts. These findings have encouraged investigations examining the treatment of islets with compounds that have antioxidant and anti-inflammatory properties in order to enhance islet viability and function post-transplant. Our study suggests that *in vitro* treatment with C3G-rich extracts of Chinese bayberry fruit improves the viability of human islets when exposed to cellular stressors that mimic the post-transplant environment. C3G-treated islets exposed to amylin or soluble oligomeric Aβ_1-42_ had increased survival and decreased amyloid-like plaque formation. Treatment of human islets with C3G also appeared to offer some protection against amylin- and Aβ_1-42_-induced ROS formation. In addition, C3G treatment of human islets prior to exposure to amylin or Aβ_1-42_ enhanced the HO-1 gene expression, which is consistent with our previous findings in mouse and pig islets. HO-1 is among the most critical components of cellular defense system against oxidative stress-induced injury, and it is thought to play a key role in maintaining antioxidant/oxidant imbalance [43]. A number of studies in islets showed that HO-1 induction significantly reduced apoptosis and promoted islet graft survival [44, 45]. Although not approaching statistical significance, stressed islets that were exposed to C3G demonstrated a greater ability to secrete insulin in glucose-rich environments than islets that were not exposed to C3G. These findings indicate that treatment of islets prior to transplantation with a protective compound such as C3G may allow for increased viability and function of the islet allograft. This could lead to an increase in the lifespan of the transplant *in vivo* and has the potential to minimize the need for strong immunosuppressive drug regimens, which have also been implicated as a contributing factor in islet transplant failure.

As mentioned, this study included an investigation of autophagy marker LC3 in an attempt to gain insight into the mechanism of C3G’s apparent protective effects against cellular stress *in vitro*. Autophagy was examined over other possible mechanisms of protection, as it has been implicated to play a critical role in the preservation of beta cells under stress [46] and is relatively easy to quantify using cellular markers like LC3, a microtubule-associated protein that is present in the cell membrane of autophagic vesicles. LC3 is therefore commonly used as a marker for measuring autophagy-related processes [47]. Findings of this study show that C3G increases the presence of LC3 protein in human islets exposed to amylin, Aβ_1-42_, H_2_O_2_, and rapamycin, which may suggest that C3G increases autophagic activity within the islet cells. This suggests an explanation for the increased viability of islets exposed to C3G. Other research in the field has reported that anthocyanin also protected rat insulinoma cell line (INS-1) against H_2_O_2_-induced cell death (necrosis and apoptosis) [32] but causes a decrease in autophagic markers [48], contrary to what we have observed in human islets. We believe that this discrepancy could be explained by the fact that Zhang et al used a rat β cell line that has been manipulated to express LC3. It is possible that these differences in experimental conditions do not elicit the same cellular stress response and therefore stimulate autophagic pathways in the cell differently.

Given the present findings, it seems likely that exposure to C3G increases the cell’s ability to undergo selective autophagy. Selective autophagy allows the cell to target specific substrates for degradation, and has been implicated in maintaining intracellular homeostasis [49]. This contrasts with non-selective autophagy, wherein cellular constituents are randomly sequestered into autophagic vesicles and destroyed, leading in cellular damage or cell death [50]. An increase in selective autophagy, suggested by the increase in LC3, would enable the islets to undergo cellular repair and regeneration in response to cellular stress, and would explain the increased viability seen in this study. However, it is unknown if the increase in autophagic markers point to an increase in autophagic vesicle formation or a decrease in autophagic vesicle destruction. Our TEM imaging data suggest the former, as an increase in vesicle formation can be seen in islet cells that have been treated with C3G. Further studies examining the time-dependent formation and destruction of autophagosomes may provide additional insights.

Additional findings from this study show that C3G significantly decreased the protein expression but (not gene and secreted products) of pro-inflammatory marker IL-1β and NLRP3. This supports the notion that an augmentation of viability and function of human islets may also occur via the anti-inflammatory effects of C3G. It is worth noting that there have been *in vivo* studies that have successfully shown prolonged survival of islet transplants via an anti-inflammatory mechanism. For example, Nasr et al showed that co-transplantation of autologous mesenchymal stem cells with human islets delayed islet transplant rejection in some recipients and corresponded to decreased immune cell proliferation and inflammatory markers [51, 52]. Although an *in vitro* examination of the effects of C3G has its limitations, these observations provide an impetus to pursuing *in vivo* studies. Such studies would include the evaluation of whether pre-treatment of islets with C3G could significantly improve the survival of islet allografts co-transplanted with mesenchymal stem cells (i.e., ≥50% of recipients with ≥100 days post-transplantation graft survival), in which C3G provides protection during islet isolation and at early time points post-transplantation and the latter provides a local immune-privileged site.

## Supporting information

S1 File Supplemental files for all results presented in the manuscript. (XLSX)
